# Growth enhancement of porcine epidemic diarrhea virus (PEDV) in Vero E6 cells expressing PEDV nucleocapsid protein

**DOI:** 10.1371/journal.pone.0212632

**Published:** 2019-03-06

**Authors:** Benjamas Liwnaree, Jaraspim Narkpuk, Suttipun Sungsuwan, Anan Jongkaewwattana, Peera Jaru-Ampornpan

**Affiliations:** Virology and Cell Technology Laboratory, National Center for Genetic Engineering and Biotechnology (BIOTEC), National Science and Technology Development Agency (NSTDA), Pathum Thani, Thailand; New York Blood Center, UNITED STATES

## Abstract

More recently emerging strains of porcine epidemic diarrhea virus (PEDV) cause severe diarrhea and especially high mortality rates in infected piglets, leading to substantial economic loss to worldwide swine industry. These outbreaks urgently call for updated and effective PEDV vaccines. Better understanding in PEDV biology and improvement in technological platforms for virus production can immensely assist and accelerate PEDV vaccine development. In this study, we explored the ability of PEDV nucleocapsid (N) protein in improving viral yields in cell culture systems. We demonstrated that PEDV N expression positively affected both recovery of PEDV from infectious clones and PEDV propagation in cell culture. Compared to Vero E6 cells, Vero E6 cells expressing PEDV N could accelerate growth of a slow-growing PEDV strain to higher peak titers by 12 hours or enhance the yield of a vaccine candidate strain by two orders of magnitude. Interestingly, PEDV N also slightly enhances replication of porcine reproductive and respiratory virus, a PEDV relative in the Nidovirales order. These results solidify the importance of N in PEDV recovery and propagation and suggest a potentially useful consideration in designing vaccine production platforms for PEDV or closely related pathogens.

## Introduction

Following a large outbreak around 2010, porcine epidemic diarrhea virus (PEDV) has emerged as an eminent threat in the swine industry worldwide [1, 2]. Although PEDV can infect pigs of all ages, mortality in infected piglets aged below one week is especially high and could reach 100%. A few strategies have been employed to control PED outbreaks. For instance, feedback of PEDV infected materials to sows can induce lactogenic immunity for piglets [3, 4]. Despite being widely adopted in farms, this strategy poses serious safety concerns as contamination of other pathogens, dosage and virulence are often not well-controlled [3, 4]. Inactivated vaccines have higher safety measures but usually give less robust protection. Especially in Asian countries, antigenic variations between emerging strains (post-2010) and classical strains may have led to failure of traditional attenuated vaccines [3, 5]. These problems urgently call for updated effective PEDV vaccines. Reverse genetics technology can immensely help with creating vaccine seeds that are attenuated and carry matching antigens and bypassing laborious and time-consuming process of tissue culture adaptation. Appropriate cell culture platforms are also critical for virus production at an industrial scale. Although Vero or Vero E6 cells are widely used to propagate PEDV at the moment, improvements in titers and replication kinetics are desirable. Both better understanding of PEDV replication and pathogenesis from basic research and improvement in technologies such as reverse genetics for generation of vaccine candidates and engineered cell lines suitable for efficient propagation of selected candidates could make immense contribution to PEDV vaccine development.

PEDV is a coronavirus (CoV) with a positive-sense RNA genome of 28 kb [6]. Its genome comprises two overlapping open reading frames (ORFs) encoding two polyproteins, ORF1a and ORF1ab, and five other ORFs encoding five proteins: spike (S), ORF3, envelope, membrane and nucleocapsid (N) [7]. PEDV entry is mediated by S protein. Once inside the cells, ORF1 and ORF1ab are translated by host ribosomes and cleaved by viral proteases into non-structural proteins which are involved in subsequent viral RNA transcription and replication [8, 9]. Structural proteins are then produced, and viral assembly commences at the endoplasmic reticulum (ER)-Golgi complexes where the viral genome encapsidated by multimers of N is packaged with viral structural proteins into virions [10, 11].

CoV N is a multi-functional protein [11]. Its primary function is to organize the viral genome and help in the viral assembly process [10]. Several lines of evidence suggest that N is required for optimal CoV RNA transcription and/or replication. First, CoV N proteins may act as RNA chaperones [12, 13]. Second, presence of N enhances recovery of several CoVs from infectious RNA, implying early roles of N during RNA synthesis [14, 15]. Third, for murine hepatitis virus (MHV) and severe acute respiratory syndrome virus (SARS-CoV), N is found to co-localize and/or interact with replicase components, possibly tethering viral RNA to the replicase complex for efficient viral RNA production [16, 17]. For transmissible gastroenteritis virus (TGEV), N is not essential for RNA replication but is required for efficient transcription [13]. Roles of PEDV N during viral RNA synthesis have not been as extensively studied but are assumed to be similar.

Besides its function in genome management, CoV N is shown to modulate cellular processes such as cell cycle, translation suppression and host immune response. Particularly for PEDV, N has been reported to induce ER stress [18]. Through interaction with cellular protein nucleophosmin, PEDV N was able to protect cells from induced apoptosis [19]. PEDV N has been shown to inhibit interferon-β (IFN-β) production and interferon-stimulating gene (ISG) expression via suppression of IFN regulatory factor 3 (IRF3) [20]. In our previous work, we demonstrated that, in some PEDV strains, N is an unusual substrate of PEDV 3C-like protease (3Cpro). Notably, we observed growth retardation of a cell culture-adapted PEDV-AVCT12 strain carrying the cleavage-resistant mutation in its nucleocapsid gene [21]. These data together suggest layers of complexity and multiple roles that PEDV N might be involved during the course of PEDV infection and consequently affect PEDV growth kinetics.

In this study, we investigated the impact of PEDV N on *in vitro* viral recovery and growth kinetics in tissue culture systems and assessed its potential benefit to PEDV vaccine production platforms. We showed that PEDV N expression can significantly enhance PEDV recovery from infectious DNA clones. Moreover, we demonstrated higher growth characteristics of some PEDV strains, including a slow-growing variant and a vaccine candidate, in Vero E6 cells expressing PEDV N. Interestingly, replication of porcine reproductive and respiratory virus (PRRSV) but not influenza virus is slightly enhanced by PEDV N. Results from this study confirm the notion that N is vital for PEDV replication and suggest a potential in adopting Vero E6-N cells as an alternative system for enhancing propagation of PEDV vaccine strains and possibly other related viruses.

## Materials and methods

### Biological materials

Human embryonic kidney (HEK) 293T cells and Vero E6 cells and their derivatives are maintained in OptiMEM supplemented with 10% Fetal Bovine Serum (FBS) and antibiotics at 37 ºC with 5% CO_2_. Plasmids for PEDV N expression (pCAGGS-PEDV N, pCAGGS-PEDV N-L381P, pCAGGS-PEDV N-Myc, pCAGGS-PEDV N-BP and pCAGSS-PEDV N-NP) were described previously [21]. The virus PEDV-AVCT12-mCherry (WT or L381P) and their infectious clones (p-SMARTBAC-PEDV_AVCT12_-mCherry [pPEDV-mCh]) were described previously [21, 22]. An infectious clone for PEDV-AVCT12_S.YN144_-mCherry was a kind gift from A. Wanitchang and A. Jongkaewwattana (unpublished results). Unless otherwise specified, the PEDV N gene used in this study was derived from PEDV strain AVCT12 (accession number LC053455).

### Rescues of reverse genetics-derived PEDV in the presence of PEDV N expression

HEK 293T cells were co-transfected with 1 μg of infectious clone and 1 μg of either PEDV N-expressing plasmid or an empty vector (FuGENE HD, Promega). At 48 h post-transfection (hpt), supernatants were transferred and adsorbed onto Vero E6 cells for 1 h at 37 ºC. The inocula were then removed, and the cells were washed twice with phosphate-buffered saline (PBS) and supplemented with 2ml OptiMEM with 0.1% TrypLE (Thermo Fisher Scientific). At indicated time points, infected cells were imaged on a fluorescence microscope (Olympus) and culture supernatants were harvested for viral RNA production analysis by RT-qPCR as described below.

#### PEDV replication in Vero E6 cells transiently expressing PEDV N

Vero E6 cells were transfected with 1 or 2 μg of pCAGGS-PEDV N-Myc (FuGENE HD, Promega). The transfection mixture was supplemented with pCAGGS vector to 2 μg plasmid to control for the total amount of DNA and transfection reagents. At 24 hpt, transfected cells were washed twice with PBS and adsorbed with PEDV-AVCT12-mCherry in OptiMEM at multiplicity of infection (MOI) of 0.0001 for 1 h. Infected cells were washed once with PBS and supplemented with 2ml OptiMEM with 0.1% TrypLE. At 48 h post-infection (hpi), culture supernatants were harvested for viral RNA production analysis by RT-qPCR as described below.

### Construction of Vero E6-N cells

The encoding region of PEDV N was amplified from pCAGGS-based plasmids and assembled into the pSIN-CSGW-UbEm lentivitral vector. The lentiviruses encoding PEDV nucleocapsid proteins were recovered based on the method described previously [23]. Briefly, HEK293T cells were transfected with lentiviral vectors in combination with a packaging plasmid (pCMV-ΔR8.91 encoding the packaging proteins Gag-Pol, Rev, Tat) and an envelope expression plasmid (pMD.G encoding the vesicular stomatitis virus glycoprotein). Viral supernatant was harvested at 48 h and filtered through a 0.45 μm filter. Subsequently, the lentiviruses were transduced into Vero E6 cells. Single clones of Vero E6 cells expressing PEDV N were identified and selected for clonal expansion. Cell proliferation and viability were followed in a resazurin-based assay [24]. Briefly, Vero E6 and Vero E6-N cells were seeded at 2x10^4^ cells/well in 96-well plates and left to grow at 37 ºC with 5% CO_2_. At indicated time after seeding, 3.125 μg resazurin was added to each well, and the plates were then incubated at 37 ºC for 6 h. Fluorescence signal is measured using SpectraMax M5 multi- detection microplate reader (Molecular Devices, USA) at the excitation and emission wavelengths of 530 nm and 590 nm. Values shown were averages±SEM from three biological replicates (each with four technical replicates).

### Virus infection

For PEDV, Vero E6 or Vero E6-N cells were infected with indicated viruses at MOI of 0.0001. One h post-adsorption, the cells were washed with PBS prior to addition of 2 ml of fresh Opti-MEM containing 0.1% TrypLE. At indicated time points, extent of PEDV replication was monitored by mCherry expression as these PEDVs contained an mCherry gene in their genomes [22]. Additionally, supernatants were collected at indicated time points for quantitative viral replication analysis by RT-qPCR or TCID_50_ assays.

To monitor replication of other viruses in the presence of PEDV N, Vero E6 and Vero E6-N cells were infected with influenza virus (IAV; A/Puerto Rico/8/34) at MOI 0.001 or porcine respiratory and reproductive syndrome virus (PRRSV; MLV Ingelvac vaccine strain) at MOI 0.01. At 36 hpi, cell lysates were harvested for protein analysis and viral RNA was extracted for viral quantification.

### TCID_50_ assay

Monolayers of Vero E6 cells in 96-well plates were adsorbed for 1 h with 100 μl of 10-fold serially diluted virus (8 wells per each dilution). After removal of inocula, the cells were washed with PBS and 200 μl of fresh Opti-MEM with 0.1% TrypLE was added into each well. At 72 hpi, the infected cells were scored by mCherry expression under a fluorescence microscope. TCID_50_ titers were calculated using the Reed-Muench method [25].

### RT-qPCR

For viral progeny quantification, viral RNA was extracted from 200 μl supernatant using a viral nucleic acid extraction kit (Geneaid, Taiwan) at indicated time points. One-step RT-qPCR was performed using an iTaq Universal SYBR Green One-Step Kit (BioRad, USA) in a 20-μl reaction with 5 μl of the extracted RNA as a template in a CFX96 Thermal cycler with the following condition: 50°C, 30 min; 95°C, 1 min; and 40 cycles of [95°C, 15 s; 60°C, 60 s]. For PEDV, primers were specific to PEDV M gene as previously described [26]. For other viruses, primers were specific to the M gene in the IAV genome (fwd, 5’-TAACCGAGGTCGAAACGTA and rev, 5’-GCACGGTGAGCGTGAA; [27]) or the N gene in the PRRSV genome (fwd, 5’-TGCCAGATGCTGGGTAAGAT and rev, 5’-TAAAGGCGGTCTGGATTGAC). Serial dilutions of pMD-PEDV M, pHW2000-PR8 M or pBS-VR2332, which contains the genome of the parent vaccine strain PRRSV VR2332, were used to generate standard curves for relative quantification of genome equivalents of viral RNA. Data were analyzed with CFX Manager software.

For analysis of viral RNA synthesis, total RNA was extracted from Vero E6 or Vero E6-N cells infected with PEDV-AVCT12-mCherry (MOI = 0.0001) at indicated time points using the RNA extraction kit (Thermo Scientific). DNaseI (Fermentas) was used to treat the RNA (15 minutes at 37 ºC) and was inactivated with EDTA (10 minutes at 65 ºC). One-step RT-qPCR was performed as described above, except with Luna Universal One-Step RT-qPCR mix (New England Biolabs). To quantify the levels of genomic RNA, we used primers specific to ORF1 (fwd: 5’-AGTACGGGGCTCTAGTGCAG and rev: 5’-GCTTATCCAAATTCTTCAGGCG; [28]). To quantify the levels of sgmRNA, we used the primer specific to the 5’UTR (fwd: 5’-AGACCTTGTCTACTCAATTCAACT; [28]) and the primer specific to the coding region of the AVCT12-S gene (rev: 5’-TAACCACCCAAAACGACGAC). The viral RNA levels were normalized to those of GAPDH mRNA (fwd: 5’-TCAACAGCGACACCCACTC and rev: 5’-CTTCCTCTTGTGCTCTTGCTG; [28]). Relative quantities of RNA accumulation were evaluated using the 2^–ΔΔCt^ method with the normalized viral RNA level from infected Vero E6 cells at 12 hpi set to one. Values are averages±SEM from three independent experiments.

### Western blotting

Transfected or infected cells were harvested at indicated time points in RIPA buffer [50 mM Tris-HCl pH 7.4, 150 mM NaCl, 1% Triton-X 100, 0.5% Na-deoxycholate, 0.1% SDS] and separated in a 10% SDS-polyacrylamide gel. PEDV N was detected with a mouse monoclonal anti-PEDV N antibody (SD 6–29, Medgene Labs) or a mouse monoclonal anti-Myc antibody (Thermo Scientific). PEDV S was detected with a mouse monoclonal anti-S1 antibody (a kind gift from Q. He, Huazhong Agricultural University). IAV nucleoprotein was detected with a mouse monoclonal anti-NP antibody (Southern Biotechnology). PRRSV N protein was detected with a rabit polyclonal anti-ORF7 antibody (Median Diagnostics). Loading control, β-actin, was detected with a mouse monoclonal anti-β-actin antibody (Cell Signaling Technology).

### Immunofluorescence assay

To study the effect of transient PEDV N expression on PEDV growth, Vero E6 cells were seeded on cover slips to reach 70% confluence on the next day. Vero E6 cells were transfected with 1 or 2 μg of pCAGGS-PEDV N-Myc (FuGENE HD, Promega). The transfection mixture was supplemented with pCAGGS vector to 2 μg plasmid to control for the total amount of DNA and transfection reagents. At 24 hpt, transfected cells were washed twice prior to adsorption with PEDV-AVCT12-mCherry (MOI = 0.01) for 1 h. Cells were then washed once and supplemented with 2ml OptiMEM with 0.1% TrypLE. At 36 hpi, cells were washed twice with PBS and fixed with cold 4% paraformaldehyde in PBS at 4˚C for 20 min. Fixed cells were washed 3 times with PBS and permeabilized with 0.2% Triton-X in 1% bovine serum albumin (BSA)+10% FBS in PBS at room temperature for 1 h. After PBS wash, cells were incubated with 1:500 primary antibodies [rabbit IgG anti-Myc (Abcam) and mouse IgG anti-S1] for 2 h, washed 5 times with 0.05% tween-20 in PBS, and stained with 1:1000 secondary antibodies [Goat anti-rabbit IgG-Alexa Flour 488 (Abcam) and anti-mouse IgG-Alexa Flour 647 (Abcam)] in dark for 1 h, washed 5 times and finally mounted on a glass slide with Prolong Gold Antifade mountant with DAPI (Invitrogen). Fluorescence images of stained cells were taken on florescence microscope (Olympus lx73).

To determine PEDV N localization, engineered Vero E6 ells were seeded on chamber slides. At 24 h, cells were fixed and permeabilized with 100% ice-cold acetone for 5 min and blocked with PBS supplemented with 1% BSA+10% FBS. After 1-h incubation with anti-PEDV N antibody (SD 6–29), permeabilized cells were washed with PBS supplemented with 0.05% tween-20. DyLight594-conjugated goat-anti-mouse IgG was then added (Thermo Scientific). After five washes, the fluorescence images were taken on fluorescence microscope. Nuclei were stained with DAPI.

### Statistical analysis

Data were prepared and analyzed with KaleidaGraph version 4.1.3 (Synergy Software). Statistical significance were determined for each pair of experimental and control groups by Student’s *t*-test. Single and double asterisks represent *p*-values of < 0.05 and <0.01, respectively. No asterisk indicates *p*-values of > 0.05.

## Results

### PEDV N expression enhances plasmid-based reverse-genetics rescue of PEDV

For different CoVs, there have been conflicting evidence whether nucleocapsid protein expression is required for viral recovery from infectious DNA or RNA [29]. The reverse genetics system for PEDV established in our laboratory did not include an extraneous N-expressing plasmid [22]. However, capped PEDV N transcripts were included during recovery of PEDV PC22A from *in vitro*-transcribed full-length PEDV RNA [30]. The effect of augmented PEDV N expression during *in vitro* recovery in the bacterial artificial chromosome (BAC)-based system has not been documented. To test this, we co-transfected HEK 293T cells with a pCAGGS plasmid expressing either PEDV N (WT), PEDV N-L381P or no protein (vec) together with pPEDV-mCh, the infectious clone for PEDV-AVCT12-mCherry, and subsequently detected the recovered progenies in Vero E6 cells. This infectious clone contains the mCherry coding sequence in place of ORF3 [22], therefore allowing us to easily follow PEDV replication by fluorescence detection. The reason we chose to investigate both of these PEDV N variants was based on our previous work on 3Cpro-induced PEDV N processing. We noticed that recovery from infectious clone and growth kinetics of the laboratory-adapted PEDV carrying 3Cpro-sensitive N (WT) in cell culture were consistently more efficient than the mutant carrying 3Cpro-resistant N (L381P) [21]. Therefore, we suspected that molecular difference in PEDV N protein might have contributed to efficiencies in viral recovery and growth.

**Fig 1:**
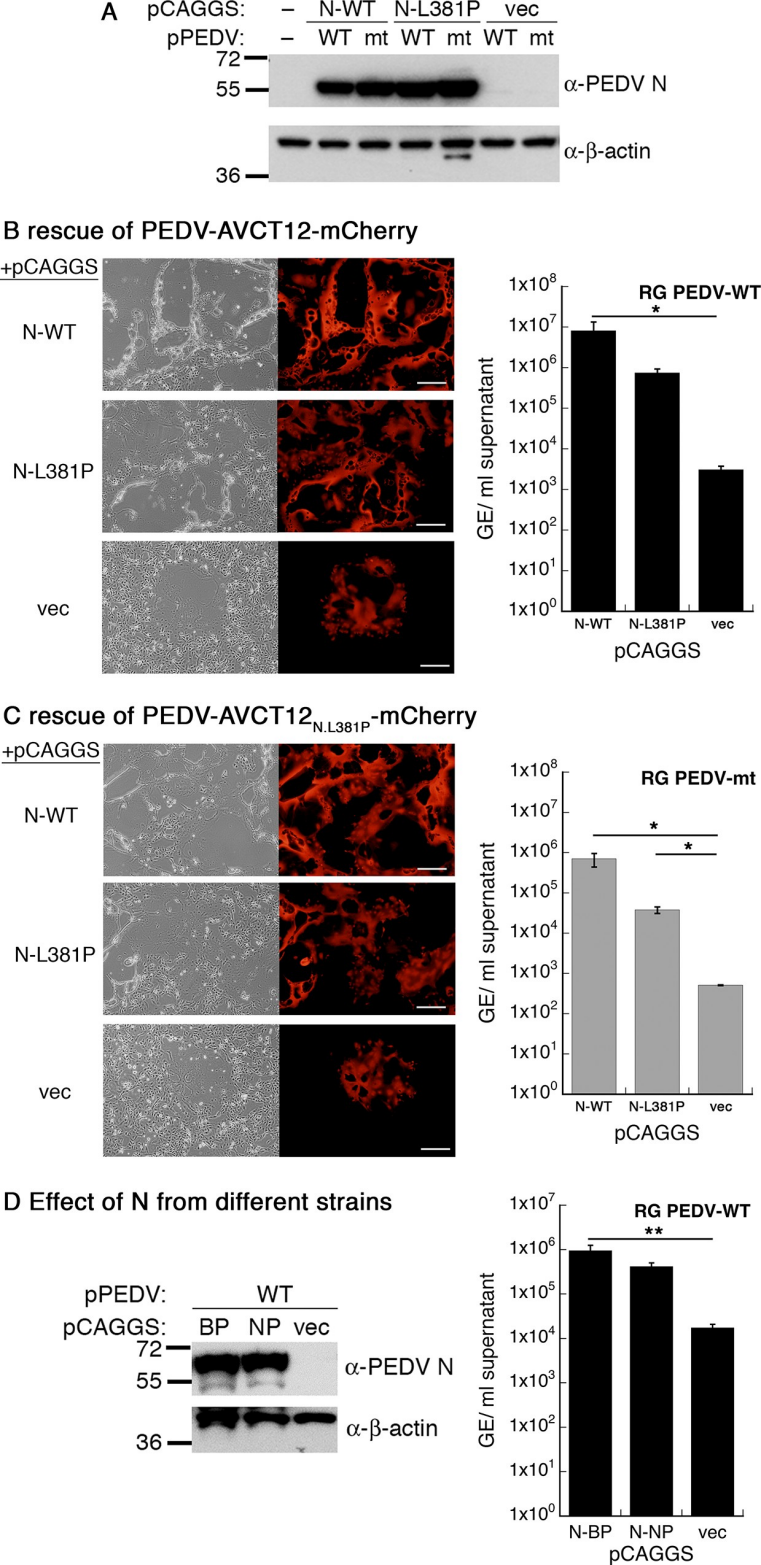
PEDV N expression enhances reverse-genetics rescue from pPEDV-mCh. HEK 293T cells were co-transfected with pPEDV-mCh (RG PEDV-WT) or pPEDV-mCh_N.L381P_ (RG PEDV-mt) and pCAGGS-N (or an empty vector). At 48 hpt, cells were lysed and analyzed by Western blotting with anti-PEDV N antibody (A) and supernatants were harvested and adsorbed onto Vero E6 cells. At 48 hpi, fluorescence images were taken to monitor replication of reverse genetics-derived PEDV-mCh (B) or PEDV-mCh_N.L381P_ (C) in Vero E6 cells. Supernatants were harvested for viral titer determination by RT-qPCR with M-specific primers (B and C). Experiments were repeated with pCAGGS expressing PEDV N derived from field strains (D). Values are averages±SEM of three independent experiments. GE, genome equivalents. * *p* < 0.05, ** *p* < 0.01.

During co-transfection in HEK 293T cells, similar expression of wild-type and L381P nucleocapsid proteins was observed from pCAGGS, while the PEDV infectious clones produced N at the level too low for detection by Western blotting (Fig 1A). Due to the low numbers of reverse genetics-derived particles, we amplified the viral progenies by passaging once in Vero E6 cells. We qualitatively monitored viral replication by the extent of mCherry expression and syncytium formation and quantified the released PEDV particles at 48 h by RT-qPCR. PEDV N expression during the rescue phase significantly enhanced virus replication as reflected by exhaustive mCherry-positive syncytia and increased the number of PEDV-AVCT12-mCherry viral progenies by three orders of magnitude (Fig 1B). Interestingly, expression of N-L381P from the plasmid also increased the numbers of the viral progenies compared to the negative control, but the effect was not as large as that observed with the wild-type PEDV N (Fig 1B). We next tested rescues of PEDV-AVCT12-mCherry carrying the L381P mutation in its N gene in the presence of absence of PEDV N expression. Consistent with previous observations, reverse genetics rescue of PEDV-AVCT12_N.L381P_-mCherry was less efficient than its wild-type counterpart. Expression of both forms of PEDV N during the reverse genetics rescue step could significantly enhance subsequent syncytium formation and raise the numbers of PEDV-AVCT12_N.L381P_-mCherry viral progenies (Fig 1C). Note that the numbers of the mutant viruses were still lower than their wild-type counterparts from the same conditions (Fig 1B and 1C, bar graphs). This might be due to an inherent growth disadvantage of the virus carrying the L381P mutation during the growth phase in Vero E6 cells.

Next, we asked if the rescue efficiency could still be improved if the N protein expressed *in trans* was not matching the strain being rescued. To this end, we compared the viral progenies produced from pPEDV-mCh co-transfection with an empty pCAGGS plasmid with those from co-transfection with a pCAGGS plasmid expressing PEDV N derived from two field isolates from Banpong (BP) and Nakorn Pathom (NP) areas in central Thailand (See S1 Fig for sequence alignment). Upon overexpression of N proteins from field strains, an increase in PEDV-AVCT12-mCherry viral progenies was observed (Fig 1D). The results are not surprising, considering that N proteins are highly conserved among PEDV strains. These data imply a general effect of PEDV N proteins in improving rescue efficiency of a PEDV vaccine strain. Taken together, data in this section suggest that exogenous PEDV N expression enhances viral production during the reverse genetics step from an infectious DNA clone for both wild-type and the slow-growing variant.

### Vero E6 cells transiently expressing PEDV N increases PEDV replication

We projected that PEDV N overexpression not only increases viral progenies during the reverse genetics process but also promotes viral replication in susceptible cells. Therefore, we tested the growth enhancement effect in cells transiently expressing PEDV N. We transfected Vero E6 cells with either pCAGGS vector or pCAGGS expressing Myc-tagged PEDV N 24 h prior to infection by PEDV-AVCT12-mCherry and observed extents of infection visually and quantitatively. We utilized the Myc-tagged PEDV N construct to enable distinction between virally-encoded N and plasmid-derived N. For fluorescence imaging, we followed expression of PEDV S to show extent of PEDV replication, as fluorescence of the virally-expressing mCherry faded upon cell fixation.

**Fig 2:**
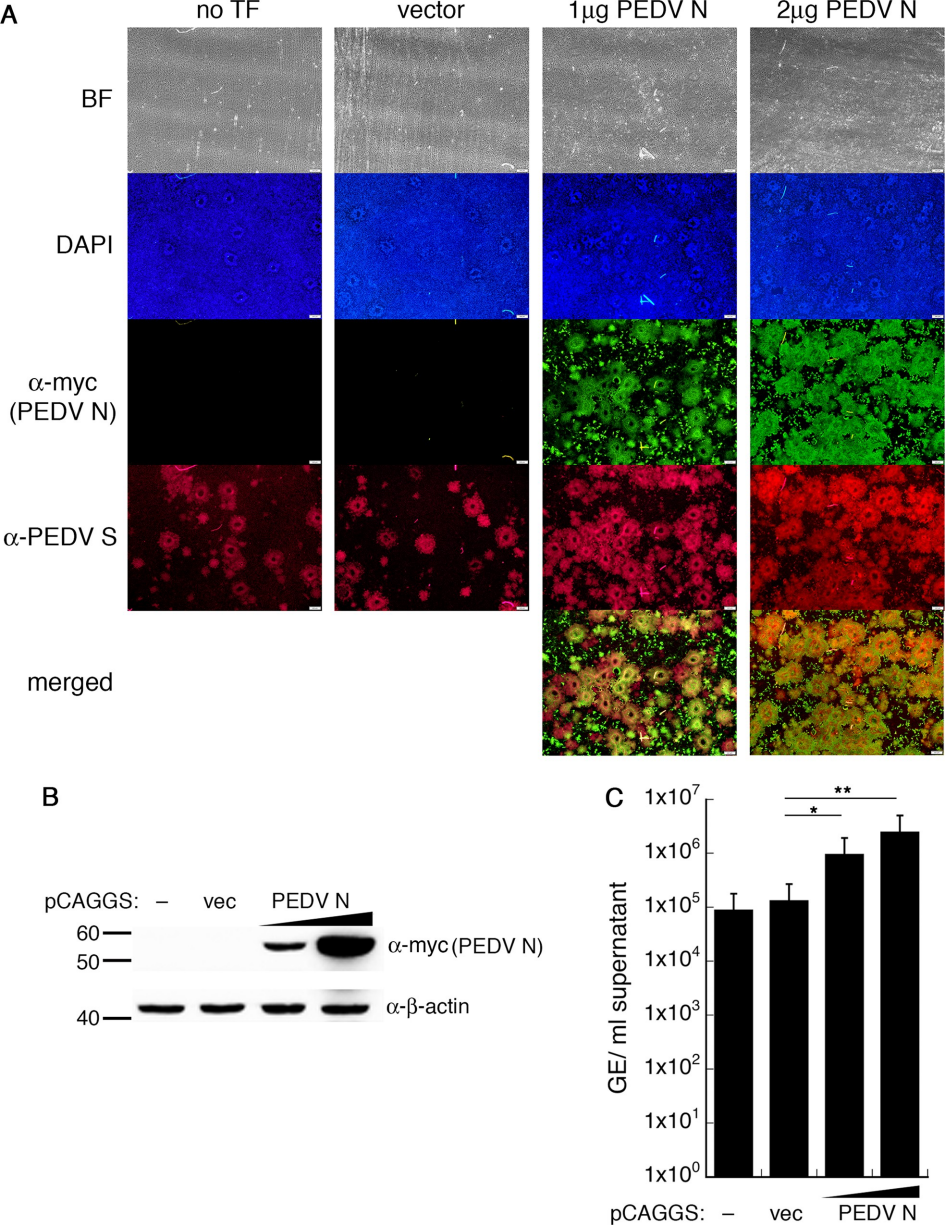
Vero E6 cells transiently expressing N improve PEDV replication. Vero E6 cells were transfected with varying combinations of pCAGGS vector and pCAGGS-PEDV N-Myc (total 2 μg). Vero E6 cells without plasmid transfection (–or no ‘TF’) served as controls. At 24 hpt, PEDV-AVCT12-mCherry were adsorbed onto transfected cells. (A) Immunofluorescence imaging of PEDV S in syncytia (red) at 36 hpi in various amounts of PEDV-N-Myc expression (green). Scale bar, 200 μm. (B) Western blot analysis of cell lysates prepared at 48 hpi. (C) RT-qPCR was performed on supernatants harvested at 48 hpi. Values are averages±SEM of three independent experiments. GE, genome equivalents. * *p* < 0.05, ** *p* < 0.01.

As indicated by the numbers of syncytia and the extent of viral spike protein produced (Fig 2A, red), Vero E6 cells pre-transfected with pCAGGS-PEDV N-Myc allowed much more profound PEDV replication at 36 hpi compared to untransfected Vero E6 cells or those pre-transfected with an empty vector. Cells pre-transfected with 2 μg of pCAGGS-PEDV N-Myc showed even more extensive syncytium formation (Fig 2A). Interestingly, most of the cells that showed extensive infection were cells that displayed PEDV N-Myc expression (Fig 2A, merged). Moreover, even though not all cells were transiently transfected, initially untransfected cells could share PEDV N-Myc from nearby cells upon cytoplasmic fusion during syncytium formation and supposedly showed the enhancement effect on PEDV replication similar to transfected cells. This argued for a direct effect of ectopic PEDV N expression on PEDV replication within the same cells rather than an indirect effect that might be exerted on bystander cells.

To complement the qualitative visual observation, we repeated the experiment in a larger infection volume and quantitatively measured viral RNA from particles released into supernatant. Western blot analysis of transfected Vero E6 cells confirmed dose-dependent expression of PEDV N-Myc upon varied amounts of the transfected plasmids (Fig 2B). PEDV titers as observed by viral RNA quantification increased in a dose-dependent manner in Vero E6 cells transiently expressing PEDV N (Fig 2C). Together, results in this section strongly suggest a role of exogenous PEDV N expression in promoting PEDV replication in cell culture system.

### Growth enhancement of PEDV variants in Vero E6 cells stably expressing PEDV N

**Fig 3:**
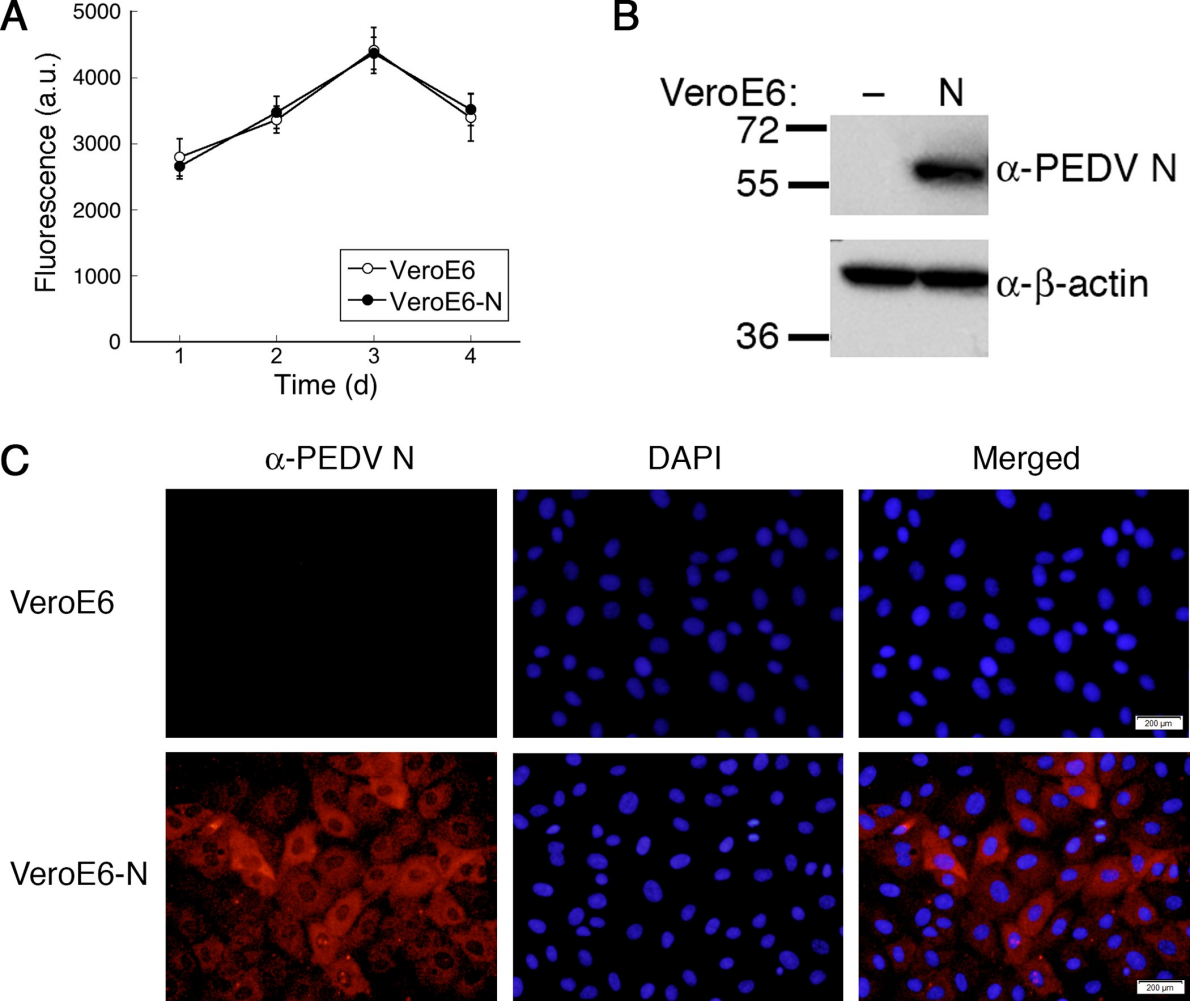
Generation of Vero E6 cells stably expressing PEDV N. Vero E6-N cells were generated by lentivirus transduction. Growth kinetics of Vero E6 and Vero E6-N cells were compared (A). PEDV N expression was analyzed by Western blotting of cell lysate (B) and immunofluorescence (C) with anti-PEDV N antibody. Nuclei were stained with DAPI. Scale bar, 200 μm.

Results from the previous sections opened up a possibility that cells expressing PEDV N might be an improved platform for PEDV seed vaccine generation. We therefore investigated the extent of PEDV replication enhancement in cells stably expressing PEDV N. First, using lentiviral vectors, we generated Vero E6 cells expressing PEDV N. After selecting single clones, growth characteristics of the engineered Vero E6 cells were compared to the original cells. In the resazurin-based viability assay, Vero E6 and Vero E6-N cells displayed similar growth kinetics, suggesting that PEDV N overexpression has no significant impact on growth of the cells (Fig 3A). PEDV N expression in these cells were verified by Western blotting (Fig 3B). PEDV N localization was characterized by immunofluorescence and was observed primarily in cytoplasm (Fig 3C). However, in some cells, PEDV N was observed as punctae inside the nuclei, suggesting that PEDV N is partially localized in nucleolus as previously observed for nucleocapsids of PEDV and other CoVs [31, 32].

**Fig 4:**
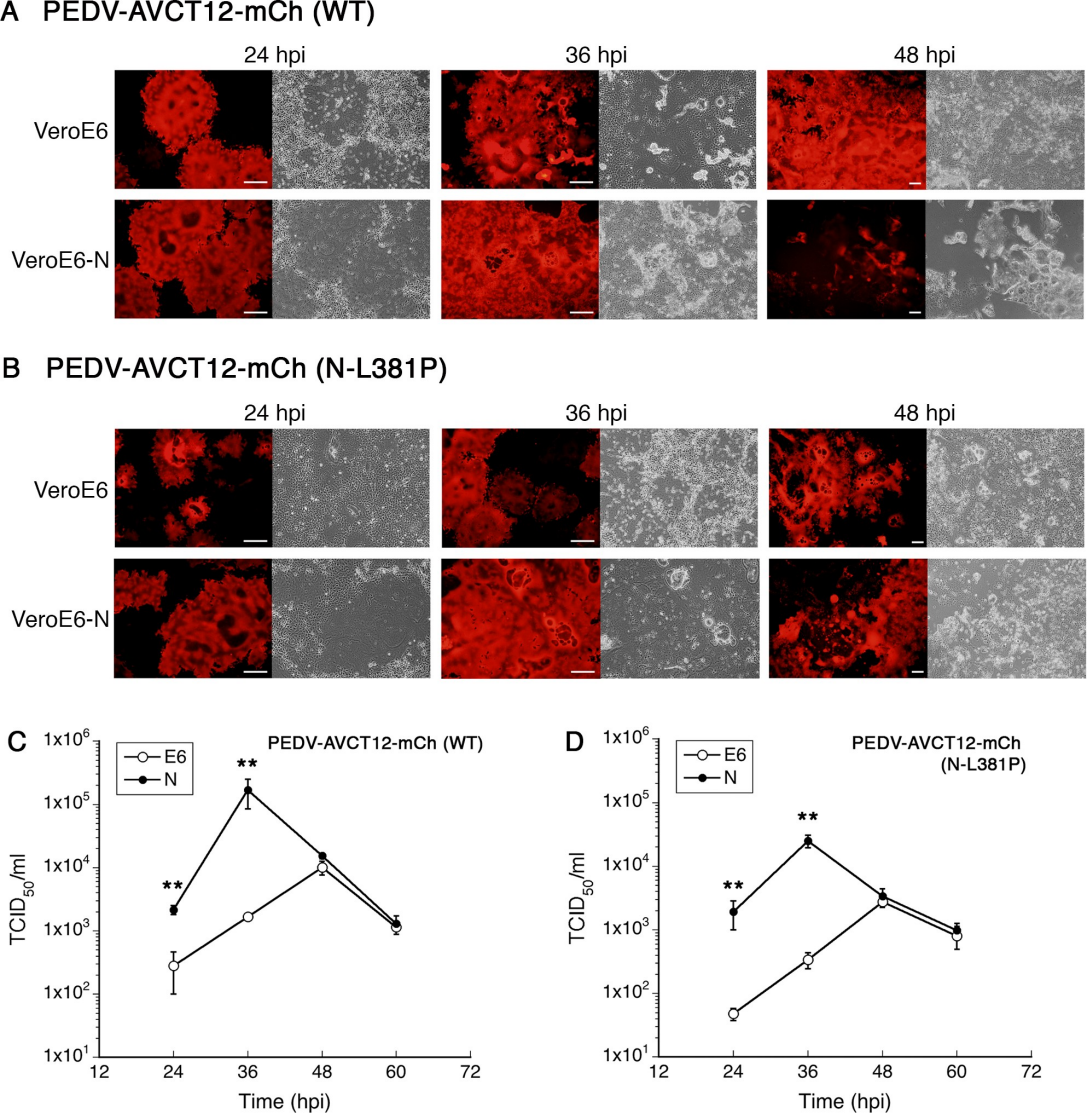
PEDV N expression in Vero E6 cells promotes growth of PEDV-AVCT12 variants. Vero E6 and Vero E6-N cells were infected (MOI = 0.0001) with PEDV-AVCT12-mCherry carrying wild-type (A and C) or L381P (B and D) nucleocapsid variants. Infected cells were imaged at indicated time (A and B), while the supernatants were analyzed for viral production by the TCID_50_ assay (C and D). Figures are representative of three independent experiments. Values are averages±SEM of three independent experiments. * *p* < 0.05, ** *p* < 0.01. Scale bar, 100 μm.

To demonstrate the effect of N expression on PEDV growth, we first studied two PEDV variants: a cell culture-adapted variant (PEDV-AVCT12-mCherry) and a slow-growing variant (PEDV-AVCT12_N.L381P_-mCherry). Equal numbers of cells were plated in 6-well plates and infected with PEDV-AVCT12-mCherry or PEDV-AVCT12_N.L381P_-mCherry (MOI = 0.0001). Low MOI was chosen for the study as it has been shown to give better production yields for some live-attenuated vaccines [33]. Infection and replication were monitored by mCherry expression at 24, 36 and 48 hpi. PEDV-AVCT12-mCherry spread much more rapidly in Vero E6-N cells when compared to infection in Vero E6 cells (Fig 4A). By 24–36 hpi, large syncytia were ubiquitously observed; by 48 hpi, almost all cells were dead and detached from the plate (Fig 4A). For the slow-growing mutant PEDV-AVCT12_N.L381P_-mCherry, very large syncytia could be easily spotted in Vero E6-N cells at 24 hpi, whereas noticeably smaller syncytia were formed in Vero E6 cells (Fig 4B). At 36 hpi, the difference was the most obvious (Fig 4B).

Quantitative assessment of PEDV replication by the TCID_50_ method also agreed with visual inspection. As expected from the qualitative visual observations, PEDV-AVCT12-mCherry replicated much faster in Vero E6-N cells, especially at early time points, with the peak titer at 36 hpi reaching mid-10^5^ TCID_50_/ml (Fig 4C). After 36 hpi, the titer dropped sharply possibly due to the pervasive cell death caused by early rapid PEDV replication such that the rate of viral production fell behind the rate of degradation of the infectious virions in the media. At 48 hpi, no significant difference in titers was observed between supernatants from the two cells. However, the virus replicating in Vero E6 cells showed a peak titer of ~10^4^ TCID_50_/ml at this time, ~10-fold lower than that observed in Vero E6-N cells at 36 hpi (Fig 4C). A similar pattern was observed for the slow-growing variant. At 36 hpi, about two orders of magnitude difference in titers was observed (Fig 4D). Even at the peak titers for the PEDV mutant grown in Vero E6 cells at 48 hpi, there still appeared to be a lag of ~an order of magnitude compared to the peak titer observed from Vero E6-N cells (Fig 4D). These data clearly demonstrate replication advantages of both PEDV variants in Vero E6-N cells and suggest that higher viral yields at a possible shorter culture time could be achieved with Vero E6-N cell platform.

### PEDV N increases viral RNA synthesis in infected cells

**Fig 5:**
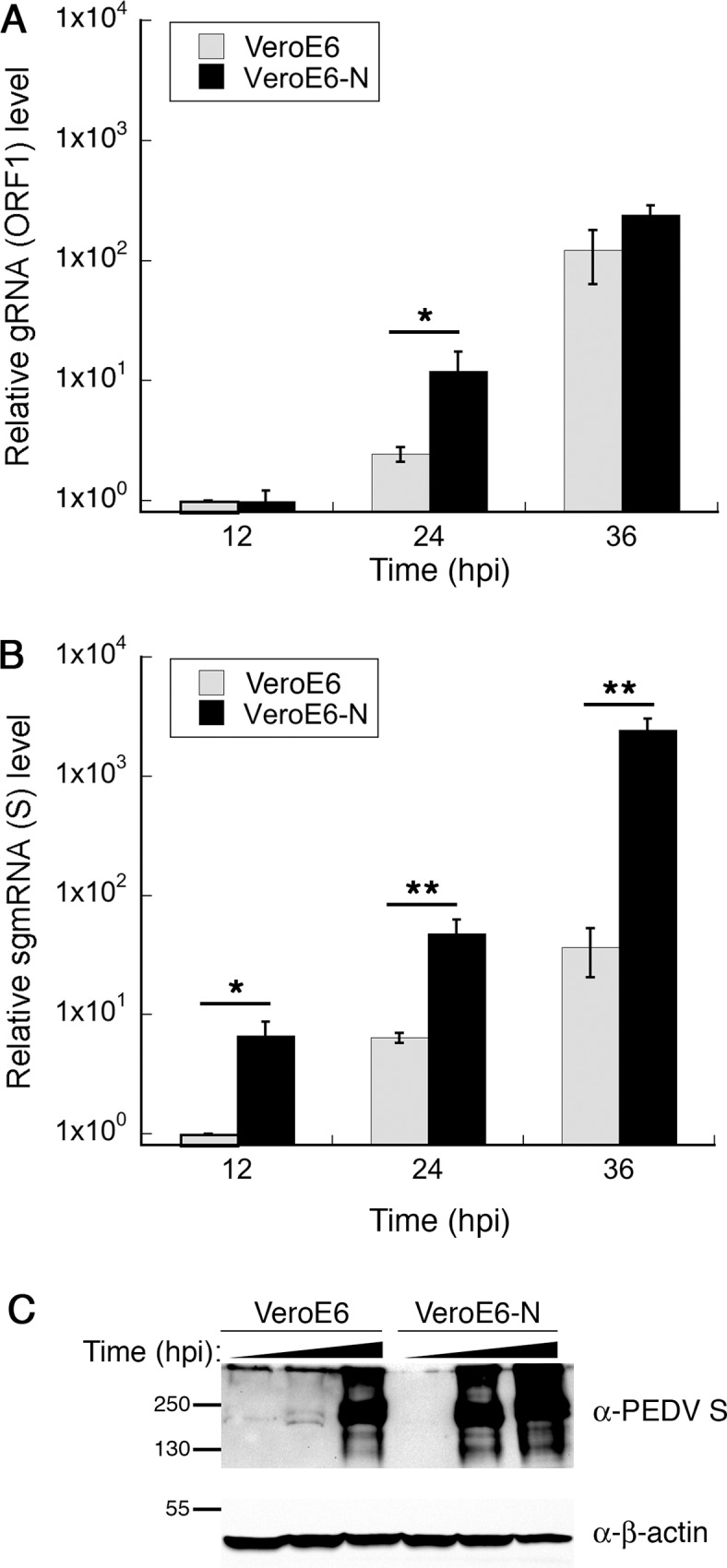
Overexpression of PEDV N enhances viral sgmRNA transcription. Vero E6 or Vero E6-N cells were infected with PEDV-AVCT12-mCherry (MOI = 0.0001) and analyzed at indicated time post-infection. For RNA analysis, total RNAs were extracted and treated with DNaseI prior to RT-qPCR experiments with primer pairs specific to ORF1 for viral RNA genome quantification (A) or to sgmRNA of S for transcription (B). Values are averages±SEM of three independent experiments. * *p* < 0.05, ** *p* < 0.01. For protein analysis, lysates were analyzed with western blotting with anti-S1 antibody (C).

To address how PEDV N-mediated increase in PEDV titer is achieved, we probed some of the mechanistic questions during viral replication. Since the most prominent functions of CoV N proteins involve viral genome management, we focused on the processes of viral RNA synthesis: transcription and replication. To measure genome replication activity, we used primers specific to ORF1 to quantify the levels of genomic RNA being produced over time. To measure transcription activity, we used the forward primer specific to the 5’UTR and the reverse primer specific to the coding region of the S gene to quantify the levels of sgmRNA. Total RNA was extracted from Vero E6 or Vero E6-N cells infected with PEDV-AVCT12-mCherry (MOI = 0.0001) at indicated time points and was used as templates for one-step RT-qPCR with indicated primers. The levels of each RNA species were normalized to the levels of GAPDH mRNA from the same conditions and were expressed as relative quantities to the RNA level at 12 hpi from infected Vero E6 cells. Vero E6-N cells showed slight increase in the levels of genomic RNA at 24 hpi, but the difference disappeared by 36 hpi (Fig 5A). On the other hand, the levels of sgmRNA in infected Vero E6-N cells were consistently and significantly higher than those observed at the same time points in Vero E6 cells (Fig 5B). Notable difference in S expression levels was also observed between Vero E6 and Vero E6-N cells (Fig 5C). Interestingly, the difference between the levels of S protein expression roughly displayed about a 12-h lag between Vero E6-N and Vero E6 cells, corroborating with similar lags observed with the levels of sgmRNA (cf. Fig 5C and 5B) and mCherry protein expression (Fig 4A). Together, these results suggest that the main mechanism through which PEDV N enhances PEDV titer is increased sgmRNA transcription and viral protein synthesis while minimally affecting genome replication activity.

### Vero E6-N cells as an alternative platform for PEDV vaccine production

**Fig 6:**
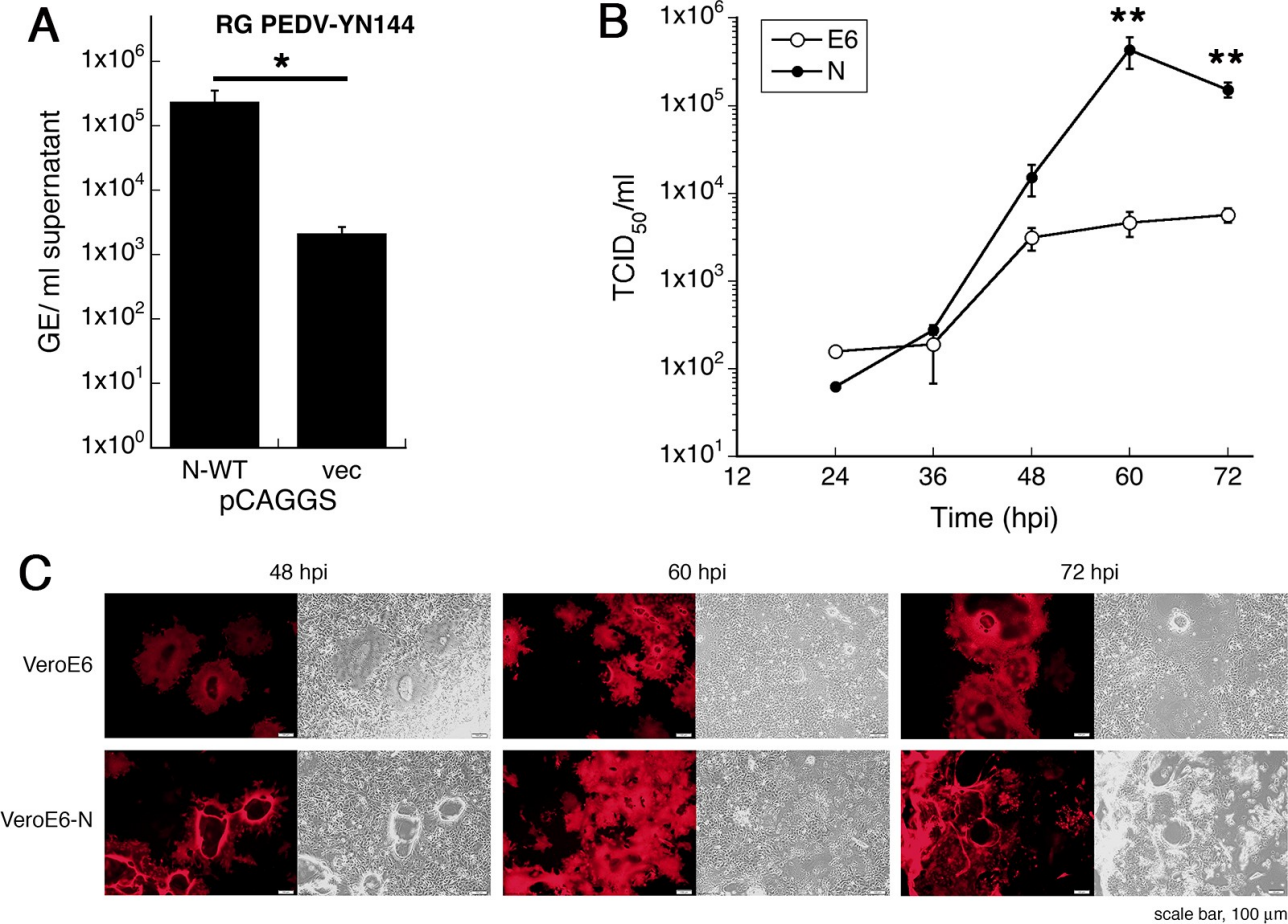
Vero E6-N cells promote replication of a vaccine candidate PEDV-AVCT12_S.YN144_-mCherry. (A) HEK 293T cells were co-transfected with pPEDV-mCh_S.YN144_ and pCAGGS-N (or an empty vector). At 48 hpt, supernatants were harvested and adsorbed onto Vero E6 cells. At 60 hpi, supernatants were harvested for viral RNA titer determination by RT-qPCR. Values are averages±SEM of three independent experiments. GE, genome equivalents. * *p* < 0.05. (B and C) Vero E6 cell variants were infected with PEDV-AVCT12_S.YN144_-mCherry (MOI = 0.0001). At indicated time points, supernatants were harvested for viral titer determination by the TCID_50_ assay (B) while infected cells were imaged (C). Values are averages±SEM of three independent experiments. ** *p* < 0.01.

To demonstrate the benefits of PEDV N overexpression on PEDV vaccine production, we tested rescue and growth of a PEDV vaccine candidate in these cells compared to the standard Vero E6 cells. Using the reverse genetics system established in our laboratory, a vaccine candidate with a spike gene derived from the strain YN144 [34] replacing the intrinsic AVCT12 spike in the PEDV-AVCT12-mCherry background (PEDV-AVCT12_S.YN144_-mCherry) was generated. This variant grew slightly more slowly than the wild-type PEDV-AVCT12-mCherry in Vero E6 cells expressing aminopeptidase N (Vero E6-APN) (A. Wanitchang and A. J., unpublished observation). First, we tested if expression of PEDV N could enhance viral recovery from an infectious clone for this particular strain. Rescue experiments were performed as described in Fig 1 but with pPEDV-mCh_S.YN144_ plasmid. RT-qPCR quantification of released particles following first-round amplification in Vero E6 cells showed significant increase in viral RNA titers if PEDV N was present during the recovery phase in HEK 293T cells (Fig 6A). We then tested whether Vero E6-N cells could help enhance replication of this vaccine candidate strain. Vero E6 and Vero E6-N cells were infected with equal amounts of PEDV-AVCT12_S.YN144_-mCherry (MOI = 0.0001), and virus replication was monitored by formation of mCherry-positive syncytium formation and the TCID_50_ assay over time. As previously observed in Vero E6-APN cells, this PEDV variant replicated noticeably more slowly than its parental strain (cf. Figs 4A and 6B). Remarkably, replication in Vero E6-N cells was enhanced by about two orders of magnitudes at peak titers (Fig 6B, 60 hpi). Visual observation of mCherry-positive syncytia also revealed more robust replication of PEDV-AVCT12_S.YN144_-mCherry in Vero E6-N cells compared to Vero E6 cells (Fig 6C). Together, data in this section suggest that overexpression of PEDV N *in trans* could help enhance efficiency of PEDV vaccine production from the reverse genetics rescue step to the viral production step.

### PEDV N slightly enhances replication of PRRSV but not influenza virus

**Fig 7:**
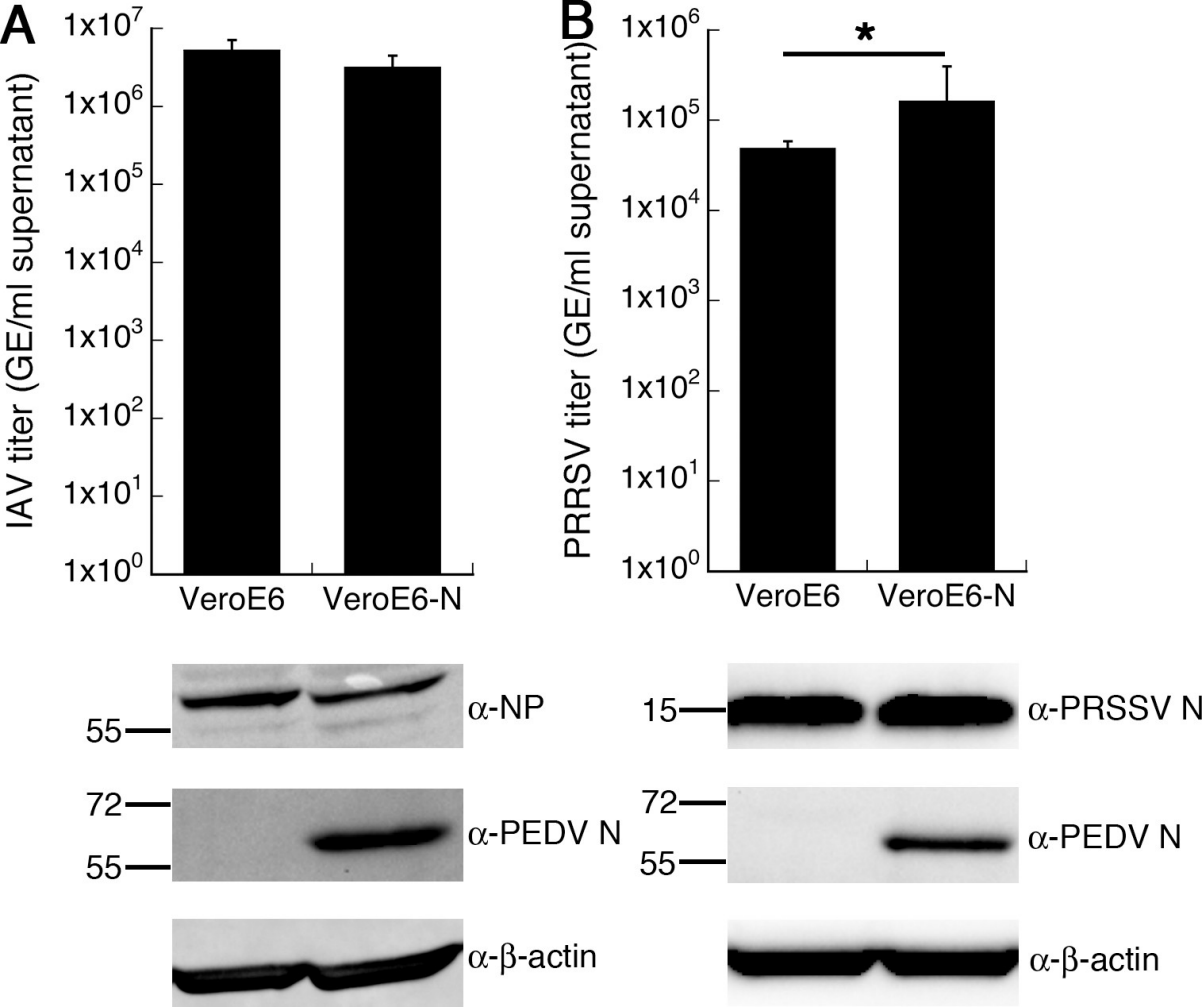
Effect of PEDV N on replication of other viruses. Vero E6 or Vero E6-N cells were infected with IAV (A) or PRRSV (B). Supernatants of infected cells were harvested for viral RNA titer determination by RT-qPCR. Values are averages±SEM of three independent experiments. * *p* < 0.05. Cell lysates were analyzed by Western blotting with indicated antibodies.

To determine if the enhancement effect exhibited by exogenous PEDV N expression is specific to PEDV or can be extended to help growth of other viruses, we examined replication of two other viruses in the presence of PEDV N. The laboratory-adapted influenza A virus (IAV, strain A/Puerto Rico/8/34) was chosen to represent a negative-sense RNA virus, while the vaccine strain of porcine reproductive and respiratory virus (PRRSV, Ingelvac MLV strain) was chosen to represent a closely-related positive-sense RNA virus. Vero E6 and Vero E6-N cells were infected with IAV (MOI 0.001) or PRRSV (MOI 0.01). At 36 hpi, infected cells were harvested for protein analysis, and supernatants were collected for released viral RNA quantification by RT-qPCR. No significant difference in IAV titers produced from the two cells could be observed (Fig 7A). On the other hand, a slight increase (< 1-log) in PRRSV RNA titers was noticed in Vero E6-N cells (Fig 7B). Western blot analysis on infected cell lyases did not reveal drastic difference in viral protein production for either virus (Fig 7). This could be due to the lower sensitivity of Western blotting compared to vRNA quantification by RT-qPCR. These results suggest that PEDV N may help slightly enhance replication of a closely-related virus such as PRRSV but have no effect on replication of an unrelated virus such as IAV. Nevertheless, it is clear that PEDV N-mediated enhancement in viral replication is much more pronounced on PEDV replication than it is on other viruses.

## Discussion

In this study, we investigated the potential that overexpression of nucleocapsid protein could promote PEDV *in vitro* viral recovery and replication kinetics in cell culture. The implication of such possibility can assist in PEDV vaccine development process. Using reverse-genetics rescue experiments, we showed that inclusion of a separate PEDV N-expressing plasmid can significantly enhance PEDV viral yields. Using Vero E6 cells engineered to express PEDV N, we demonstrated substantial replication advantages in several PEDV variants.

There have been many precedents in which nucleocapsid proteins have been shown to enhance viral recovery and propagation. For minus-strand RNA viruses, nucleocapsid proteins are required for viral RNA transcription and replication [35, 36]. Therefore, reverse genetics systems for these viruses typically include separate expression systems for their nucleocapsid proteins [37, 38]. Although theoretically not necessary, for some plus-strand RNA viruses, expression of nucleocapsid proteins is required for or can enhance viral recovery during rescue. For TGEV, recovery of virus from transfection of TGEV transcripts requires transfection of TGEV N transcripts, even though TGEV recovery from the BAC system does not exhibit this requirement [39]. Similar observations were reported in efforts to rescue other CoVs from RNA transcripts, such as infectious bronchitis virus, MHV, and SARS-CoV [14, 40, 41]. In the case of PEDV, rescue from infectious RNA was aided by inclusion of the N gene transcript [30]. However, for the BAC-based PEDV reverse genetics system, the virus could be recovered without an extraneous N-expressing plasmid [22]. In this report, we showed that exogenous N protein, although not necessary, can significantly enhance PEDV rescue titers from the BAC system. These data from multiple CoV rescue experiments demonstrate the importance of N in efficient transcription and replication of CoVs and support previous suggestions that CoV N might play essential roles during early RNA synthesis [15, 16].

Expression of N *in trans* can also improve production of infectious PEDV particles during propagation. Based on its role during the replication cycle, possible mechanisms through which PEDV N exerts its effect include viral RNA synthesis, viral particle assembly and IFN antagonism [11]. As our experiments were conducted in IFN-β-deficient Vero E6 cells, we are inclined to think that IFN antagonism is not primarily responsible for the observed enhancement in PEDV replication kinetics. Nevertheless, we could not entirely dismiss this possibility as PEDV N interferes with IRF-3 signaling [20], which could lead to suppression of other ISG responses independent of IFN-β. Indeed, it would be interesting to use other experimental systems with an intact IFN pathway to tease apart possible contribution on viral growth enhancement from PEDV N’s IFN antagonism function. On the other hand, CoV N has been shown to be critical for CoV assembly. CoV N interacts with other CoV structural proteins, especially the membrane protein, as observed in MHV, TGEV and SARS-CoV [42, 43]. Even though N is not necessary for CoV envelope formation, expression of N greatly increases yields of virus-like particles in MHV and SARS-CoV, suggesting its important role in formation of complete virions [44, 45].

As the primary role of nucleocapsid protein involves viral genome management, we directly asked if ectopic PEDV N expression affects viral RNA synthesis during infection. While genome replication activity is enhanced at an early time point, marked increase in subgenomic RNA production could be observed in Vero E6-N cells over a sustained period, leading to large excess of viral protein production (Fig 5). In this experimental system, enhancement of subgenomic transcription might be the primary mechanism of the PEDV N-mediated viral growth enhancement. Several reports have also documented important roles of nucleocapsid proteins during viral RNA synthesis. In general, CoV N can act as an RNA chaperone, facilitating the adoption of proper RNA conformation for transcription or replication [12, 13]. For TGEV, efficient replicon activity could be observed only from replicons harboring the N gene, while activity of a replicon lacking TGEV N could be rescued in cells expressing TGEV N, demonstrating that N expression either in *cis* or in *trans* is required for efficient viral RNA synthesis [46]. Replication of Human CoV-229E genomic RNA is substantially increased by expression of HCoV N and, to a lesser extent, PEDV N [47]. MHV N interacts with a replicase component and is implicated in formation of the initiation complex required for negative-strand synthesis [16, 17]. Similar interactions between N and a replicase component have been recently described in PRRSV, a member of the same Nidovirales order [48]. Indeed, structural analysis has previously indicated structural similarity between C-terminal portions of SARS-CoV N and PRRSV N and suggested a common origin between the two viral families [49]. Interestingly, while influenza virus, a minus-strand RNA virus that replicates inside the host nucleus, was unaffected by PEDV N expression, PRRSV shows slightly enhanced replication in Vero E6-N cells. This specificity also indicates that the enhancement mechanism might be more specific to the replication cycle or machinery commonly shared by Nidoviruses rather than broadly antagonizing host antiviral state. As discussed in Yount *et al*., other possible mechanisms include enhancement of viral mRNA translation or viral genome protection performed by CoV N proteins [50].

In summary, we showed here that expression of PEDV N can enhance rescue and replication kinetics of PEDV. Importantly, we observed both higher peak titers and/or shorter culture time to reach peak titers. Of course, N overexpression cannot alone solve the yield problem for every PEDV variant; viral growth in cell culture depends on multiple factors, especially spike variants and infectivity. Indeed, we could not observe any advantage in propagation of PEDV field isolates in Vero E6-N cells (B.L. unpublished results). However, in combination with other technological advancement, we might be able to harness the potential of PEDV N in improving yields for the whole chain of modern vaccine production, from rescues of vaccine candidates from the reverse genetics process to cultivation and amplification of PEDV vaccine viruses in cell culture. Although the increase might seem small, ~1–2 orders of magnitude improvement and a decrease in cultivation time to harvest at peak titers could translate to significant cost reduction during manufacturing processes.

## Supporting information

S1 Fig Alignment of PEDV N sequences used in this study. AVCT12 represents the vaccine strain, while the other two strains were field isolates from Banpong (BP) and Nakorn Pathom (NP) areas in central Thailand.
(TIFF)
