# Supplementary figures and images for: Growth enhancement of porcine epidemic diarrhea virus (PEDV) in Vero E6 cells expressing PEDV nucleocapsid protein
Source: PLoS One. 2019 Mar 6;14(3):e0212632. doi: 10.1371/journal.pone.0212632 (PMC6402621; doi:10.1371/journal.pone.0212632)

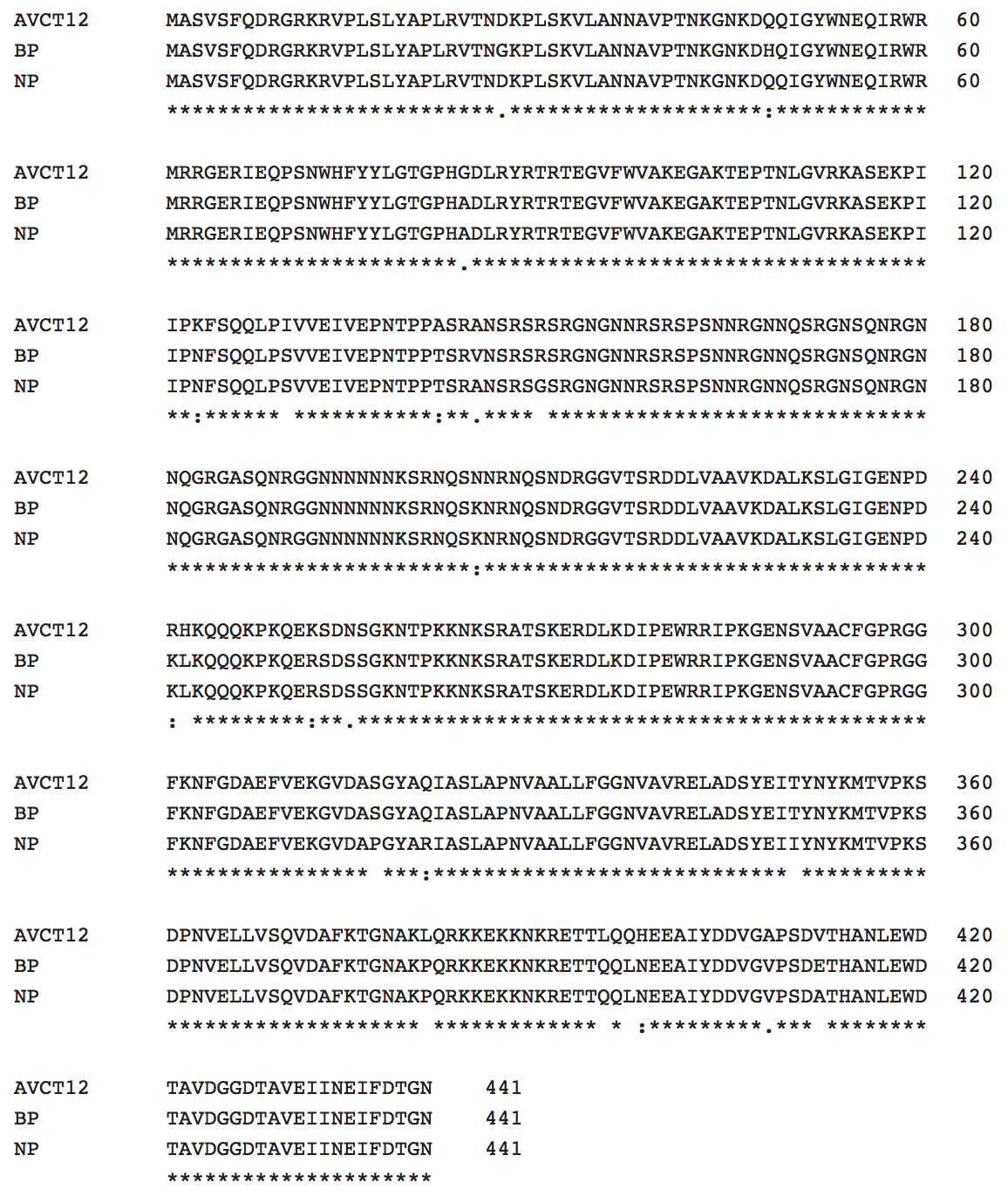

Supplement: S1 Fig — AVCT12 represents the vaccine strain, while the other two strains were field isolates from Banpong (BP) and Nakorn Pathom (NP) areas in central Thailand. (TIFF) [file pone.0212632.s001.tiff]
